# Endoscopic ultrasonography‐guided tissue acquisition for small solid pancreatic lesions: Does the size matter?

**DOI:** 10.1002/deo2.52

**Published:** 2021-09-28

**Authors:** Yousuke Nakai, Tsuyoshi Hamada, Ryunosuke Hakuta, Kazunaga Ishigaki, Kei Saito, Tomotaka Saito, Naminatsu Takahara, Suguru Mizuno, Hirofumi Kogure, Kazuhiko Koike, Mitsuhiro Fujishiro

**Affiliations:** ^1^ Department of Gastroenterology Graduate School of Medicine The University of Tokyo Tokyo Japan; ^2^ Department of Endoscopy and Endoscopic Surgery Graduate School of Medicine The University of Tokyo Tokyo Japan

**Keywords:** endoscopic ultrasound, fine needle aspiration, fine needle biopsy, pancreatic lesions

## Abstract

Endoscopic ultrasonography‐guided tissue acquisition (EUS‐TA) is now an established technique to obtain the pathological diagnosis of solid pancreatic lesions (SPLs), but the diagnosis of small SPLS by EUS‐TA can still be difficult. We conducted a literature review and a meta‐analysis on the diagnostic yield of EUS‐TA according to the tumor size. In a meta‐analysis of 33 studies with 6883 cases, a pooled odds ratio (OR) of sensitivity was significantly higher in SPLs of >20 mm (OR 1.64, *p* = 0.02) and in SPLs of >10 mm (OR 3.05, *p* = 0.01), but not in SPLs of >30 mm (OR 1.18, *p* = 0.46). The meta‐analysis of accuracy also showed a similar trend: OR of 1.59 in SPLs of >20 mm (*p* < 0.01) and OR of 3.27 in SPLs of >10 mm (*p* < 0.01) and OR of 1.03 in SPLs of >30 mm (*p* = 0.87). The use of a 25‐gauge needle tended to improve sensitivity in small SPLs, though not statistically significant: OR of 1.25 and 2.82 in studies with and without a 25‐gauge needle (*p* = 0.08). The use of fine needle biopsy needles, slow pull method, and rapid on‐site evaluation did not significantly improve sensitivity in small SPLs. EUS‐TA for small SPLs, especially neuroendocrine neoplasms, is reported to have a high risk of adverse events. In summary, the diagnostic yield and safety of EUS‐TA for small (<20 mm) SPLs still needs improvement, and the best needle and technique for small SPLs should be further investigated.

## INTRODUCTION

Endoscopic ultrasonography‐guided fine needle aspiration (EUS‐FNA), first introduced in the early 1990s,^1^, ^2^ is now established as a safe and reliable technique to obtain the pathological diagnosis of solid pancreatic lesions (SPLs). Recently, new needles to obtain histological cores, so called EUS‐guided fine needle biopsy (EUS‐FNB) needles, are increasingly used in clinical practice.^3^, ^4^, ^5^ While EUS‐guided tissue acquisition (EUS‐TA), either EUS‐FNA or EUS‐FNB, for SPLs provides high diagnostic sensitivity, there are some limitations in the diagnosis of small SPLs. It is still unknown whether new FNB needles can overcome this limitation of the tumor size or whether specific sampling technique such as the suction technique and the needle size is recommended in small SPLs. In this review, we summarize the current evidence of EUS‐TA, including a meta‐analysis of the diagnostic yield, to evaluate the effects of the tumor size on clinical outcomes of EUS‐TA for SPLs.

### Methodology and search results of a meta‐analysis of the diagnostic yield of EUS‐TA

A meta‐analysis was conducted on the diagnostic yield of EUS‐TA according to the tumor size. A systematic electronic search using MEDLINE/PubMed, Web of Science, and the Cochrane Central Register of Controlled Trials (CENTRAL) was conducted to identify clinical studies evaluating EUS‐TA for pancreatic solid lesions according to the lesion size that had been reported until May 2021. The key search words were “endoscopic ultrasonography,” “EUS‐FNA,” “fine needle aspiration,” “fine needle biopsy,” and “pancreatic neoplasms.” We included fully published articles that had involved ≥20 patients in total and limited the search to English language. The following data were extracted using a prespecified data extraction form: study design, procedure details (the needle size and type, suction methods and the number of passes, the presence of rapid on‐site evaluation [ROSE]), the diagnostic yield (adequacy, sensitivity and accuracy), and adverse events. Due to the heterogeneity of EUS‐TA procedures within each study, one factor was considered as present if that was used in some cases within the given study. For example, both FNA and FNB needles were used in some studies, and those studies were considered as studies with FNB in our meta‐analyses. Meanwhile, when procedure details were not available, those studies were excluded from the analyses. Using the data extracted from the studies identified, summary odds ratios (ORs) of adequacy, sensitivity, and accuracy according to the lesion size were computed as means of the Dersimonian‐Laird random‐effects model.^6^ The thresholds of the lesion size were set at 10, 20, and 30 mm. Given potential heterogeneity in study populations and endoscopic procedures between the studies, we utilized the random‐effects model throughout the study. Statistical heterogeneity between studies was assessed based on the *Q* and *I^2^* statistics.^7^ For the *Q* statistic, we used a *p* value of 0.10 for statistical significance in view of the low power of tests for heterogeneity.^8^ The *I^2^* statistics of around 25%, 50%, and 75% were considered as suggestive of low‐, moderate‐, and high‐level heterogeneity, respectively.^9^ We calculated 95% confidence interval (CI) for each summary OR. A two‐sided *p* value < 0.05 was considered statistically significant. All analyses were performed using R software version 3.6.3 and the *meta* package (R Development Core Team; http://www.r‐project.org) and EZR (Saitama Medical Center, Jichi Medical University, Saitama, Japan), a graphical user interface for R.^10^

**FIGURE 1:**
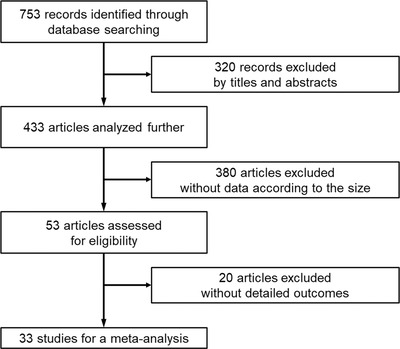
Flowchart of study selection for meta‐analysis of EUS‐guided tissue acquisition according to the size of solid pancreatic lesions

The initial search identified a total of 753 publications and after screening for eligibility, 33 studies with 6883 cases^11^, ^12^, ^13^, ^14^, ^15^, ^16^, ^17^, ^18^, ^19^, ^20^, ^21^, ^22^, ^23^, ^24^, ^25^, ^26^, ^27^, ^28^, ^29^, ^30^, ^31^, ^32^, ^33^, ^34^, ^35^, ^36^, ^37^, ^38^, ^39^, ^40^, ^41^, ^42^, ^43^ were included in the analysis: eight prospective studies and 25 retrospective studies (Figure 1). The study characteristics are summarized in Table 1, and the results of our meta‐analysis are summarized in Table 2.

**TABLE 1:** Study details

Author	Year	*n*	study design	Size, mm	Needle type	Needle size, gauge	Suction technique	ROSE	Number of pass
Williams	1999	144	Retrospective	36^†^	FNA	22, 23	Suction	NA	3.4^†^
Agarwal	2004	45	Retrospective	30.3^†^	FNA	NA	NA	NA	NA
Volmer	2005	489	Retrospective	30^†^	FNA	NA	NA	Y	3.57^†^
Ardengh	2007	405	Retrospective	34^†^	FNA	22	NA	Y	2.2^†^
Hwang	2009	139	Retrospective	40.5^†^	FNA, FNB	19, 22	Suction	N	2.7^†^
Fisher	2009	100	Prospective	35.1^†^	FNA	22	NA	Y	2.7^†^
Uehara	2011	120	Retrospective	NA	FNA	22, 25	Suction	Y	2.3^†^
Krishna	2012	232	Retrospective	NA	FNA	NA	NA	Y	NA
Haba	2013	996	Retrospective	32*	FNA	19, 22, 25	NA	Y	2*
Kim	2014	240	Retrospective	21^†^	FNA	19, 22, 25	Suction, no suction	N	3.26^†^
Sur	2015	70	Retrospective	35.2^†^	FNB	25	NA	NA	NA
Kim	2015	180	Retrospective	NA	FNA, FNB	22, 25	NA	Y	4*
Uehara	2015	117	Retrospective	23^†^	FNA	22, 25	Suction	Y	1.5^†^
Hijioka	2016	58	Retrospective	24.1^†^	FNA	19, 22, 25	Suction	Y	NA
Fujimori	2016	37	Retrospective	20.5^†^	FNA	22, 25	NA	Y	3.2^†^
Mukai	2016	82	Prospective	27.5^†^	FNA	22	Suction	NA	4
Ramesh	2016	315	Retrospective	NA	FNA	19, 22, 25	NA	Y	2.9^†^
Seicean	2016	118	Prospective	35.6^†^	FNA	22	Slow pull	N	2*
Chen	2016	102	Retrospective	34*	FNA	22	Suction, slow pull	N	3*
Malak	2016	90	Retrospective	39.5^†^	FNA	22, 25	Suction, slow pull	Y	2^†^
Mohamadnejad	2017	202	Prospective	32.5^†^	FNA	22	Suction	NA	NA
Cheng	2018	249	Prospective	NA	FNA, FNB	22	Suction, slow pull	N	NA
Ge	2018	138	Retrospective	27.6^†^	FNA	25	Slow pull	Y	3.7^†^
Yang	2018	181	Retrospective	28.89^†^	FNA, FNB	19, 22, 25	Suction, slow pull	N	NA
Sugiura	2019	788	Retrospective	NA	FNA	19, 22, 25	NA	Y	2.8^†^
Sato	2019	188	Retrospective	27*	FNA	22	Suction	N	NA
Sweeney	2020	204	Retrospective	29^†^	FNA, FNB	19, 22, 25	Suction, slow pull	Y	NA
Mizukawa	2020	97	Prospective	25*	FNA	21, 22	Suction	Y	2
Ishigaki	2020	154	Retrospective	25*	FNA, FNB	22	Suction	N	4*
Takahashi	2021	159	Retrospective	28.4^†^	FNB	22	Suction	N	2*
Teodorescu	2021	61	Retrospective	35^†^	FNA	22	Slow pull	N	4
Bang	2021	129	Prospective	NA	FNB	22	Suction, slow pull, no suction	N	NA
Ishigaki	2021	254	Prospective	29*	FNB	22	Suction, slow pull	Y	2*

Abbreviations: FNA, fine needle aspiration; FNB, fine needle biopsy; NA, not available; ROSE, rapid on‐site evaluation.

* median

^†^ mean.

**TABLE 2:** Summary odds ratios according to the size of solid pancreatic lesions

	Adequacy		Sensitivity		Accuracy	
	OR (95%CI)	*p* value	OR (95%CI)	*p* value	OR (95%CI)	*p* value
>30 mm	NA*	NA	1.18 (0.76–1.84)	0.46	1.03 (0.70‐1.51)	0.87
>20 mm	2.52 (1.80–3.53)	<0.01	1.64 (1.07–2.51)	0.02	1.59 (1.16‐2.18)	<0.01
>10 mm	NA*	NA	3.05 (1.25–7.42)	0.01	3.27 (1.55‐6.89)	<0.01

Abbreviations: CI, confidence interval; FNA, fine needle aspiration; FNB, fine needle biopsy; NA, not available; OR, odds ratio.

* Only two studies and one study reported adequacy at the threshold of 30 mm and 10 mm, respectively.

### The diagnostic yield of EUS‐TA according to the size of SPLs: A meta‐analysis

**FIGURE 2:**
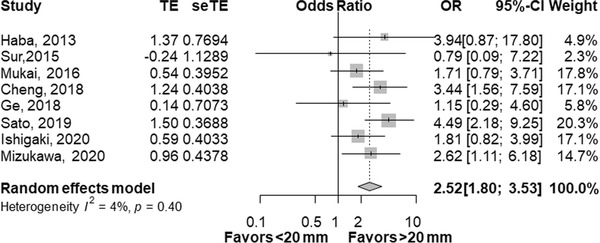
Comparison of adequacy between SPLs of <20 mm and >20 mm. Odds ratio (OR) for SPLs < 20 mm compared with SPL > 20 mm is presented for each study (center of gray square) with 95% confidence interval (CI; horizontal line). Summary OR based on a meta‐analysis via the random‐effect model is presented at the bottom of each panel (center of black diamond) with 95% CI (the width of black diamond). *p*‐value for the Q‐statistic for between‐study heterogeneity is shown

**FIGURE 3:**
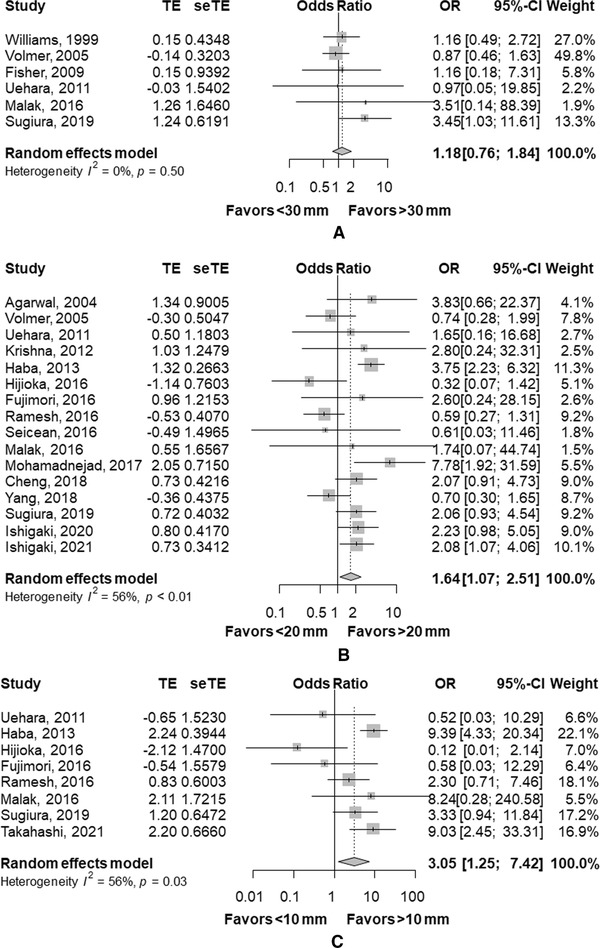
Comparison of sensitivity. (a) Comparison between lesions of <30 mm and >30 mm. (b) Comparison between lesions of <20 mm and >20 mm. (c) Comparison between lesions of <10 mm and >10 mm Abbreviations: CI, confidence interval; OR, odds ratio.

**FIGURE 4:**
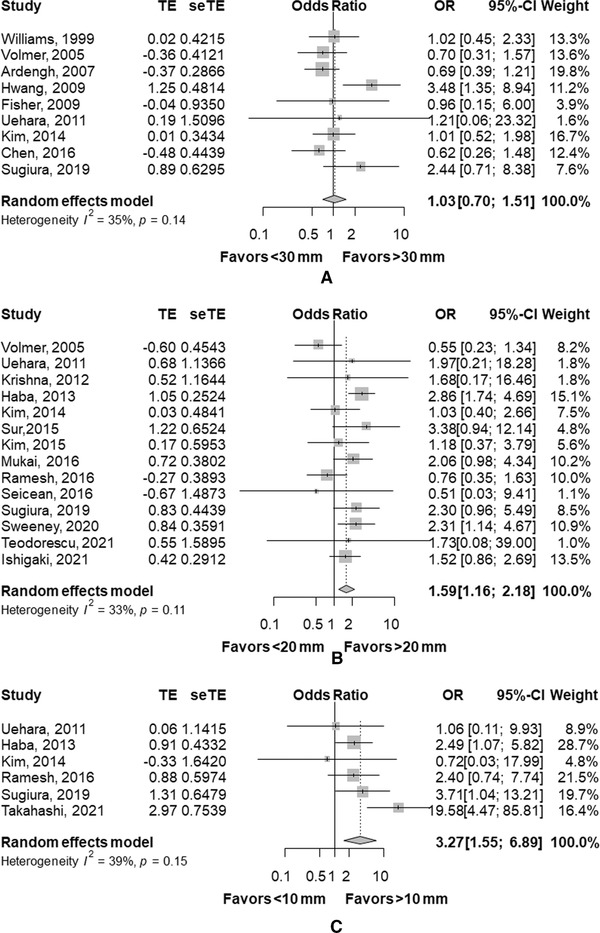
Comparison of accuracy. (a) Comparison between lesions of <30 mm and >30 mm. (b) Comparison between lesions of <20 mm and >20 mm. (c) Comparison between lesions of <10 mm and >10 mm Abbreviations: CI, confidence interval; OR, odds ratio.

There are few studies on adequacy of EUS‐TA comparing at the threshold of 30 mm (*n* = 2) and 10 mm (*n* = 1). A meta‐analysis of eight studies comparing adequacy of EUS‐TA in SPLs of <20 mm and >20 mm revealed adequacy was significantly higher in SPLs > 20 mm with an OR of 2.52 (95% CI, 1.80–3.52, *p* < 0.01, Figure 2). In terms of sensitivity (Figure 3), a pooled OR of sensitivity was significantly higher in SPLs of >20 mm (OR 1.64, 95% CI, 1.07–2.51, *p* = 0.02) and in SPLs of >10 mm (OR 3.05, 95% CI, 1.25–7.42, *p* = 0.01). However, the differences of sensitivity were not statistically significant in SPLs of >30 mm with an OR of 1.18 (95% CI, 0.76–1.84, *p* = 0.46). Similar trends were found in the analysis of accuracy (Figure 4): OR was significantly higher in SPLs of >20 mm (OR 1.59, 95% CI, 1.16–2.18, *p* < 0.01) and in SPLs of >10 mm (OR 3.27, 95% CI, 1.55–6.89, *p* < 0.01), but not in SPLs of >30 mm (OR of 1.03, 95% CI, 0.70–1.51, *p* = 0.87).

**FIGURE 5:**
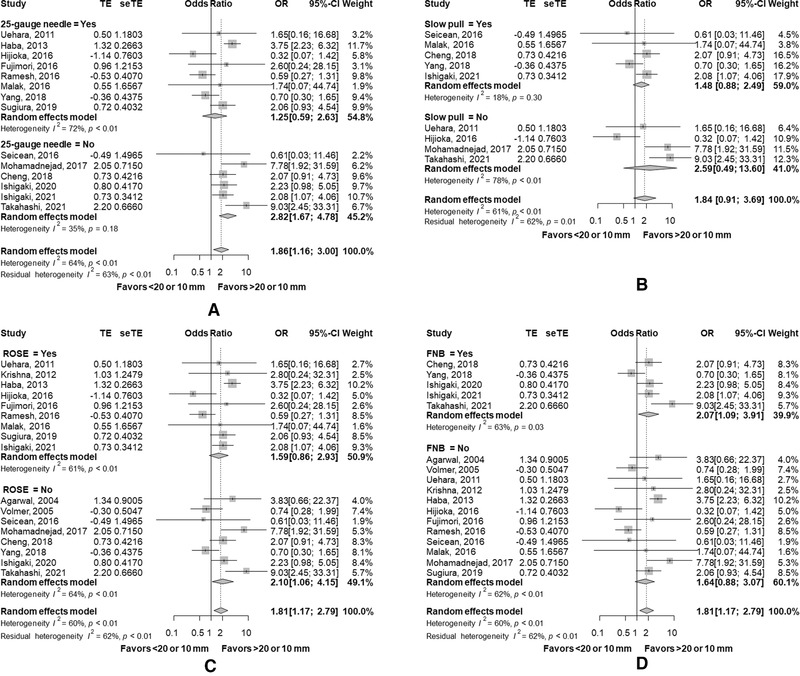
Subgroup analyses of sensitivity between small (<20 or 10 mm) and non‐small lesions. (a) Subgroups with and without 25‐gauge needles. (b) Subgroups with and without slow pull methods. (c) Subgroups with and without rapid on‐site evaluation. (d) Subgroups with and without fine needle biopsy Abbreviations: CI, confidence interval; FNB, fine needle biopsy; OR, odds ratio; ROSE, rapid on‐site evaluation.

In summary, results of our meta‐analysis revealed that the diagnostic yield of EUS‐TA for SPLs of <20 mm was inferior to that for SPLs of >20 mm. The trend was more prominent at the threshold of SPLs of 10 mm in terms of pooled ORs of sensitivity and accuracy. Exploratory analyses were also conducted to identify any technique can overcome this limitation of EUS‐TA for small SPLs. Subgroup analyses by the needle size and type, the suction and ROSE were performed for the sensitivity of EUS‐TA in small (<20 mm or <10 mm) SPLs (Figure 5). The results of subgroup analyses are summarized in Table 3. The use of FNB needles, slow pull method, and ROSE did not significantly improve sensitivity in small SPLs. The use of a 25‐gauge needle tended to improve sensitivity of EUS‐TA for small SPLs, though not statistically significant. In eight studies without a 25‐gauge needle, OR was as high as 2.82 (95% CI, 1.67–4.78), but it was 1.25 (95% CI, 0.59–2.63) in six studies with a 25‐gauge needle (*p* = 0.08).

**TABLE 3:** Subgroup analyses of sensitivity according to the needle type and the technique

		OR (95%CI)	*p* value
25‐gauge needle	Yes	1.25 (0.59–2.63)	0.08
	No	2.82 (1.67–4.78)	
Slow pull	Yes	1.48 (0.88–2.49)	0.53
	No	2.59 (0.49–13.60)	
ROSE	Yes	1.59 (0.86–2.93)	0.55
	No	2.10 (1.06–4.15)	
FNB	Yes	2.07 (1.09–3.91)	0.62
	No	1.64 (0.88–3.07)	

Abbreviations: CI, confidence interval; FNB, fine needle biopsy; OR, odds ratio; ROSE, rapid on‐site evaluation.

### The needle size

In a recent meta‐analysis of seven randomized controlled trials (RCTs),^44^ a 25‐gauge FNA needle is not superior to a 22‐gauge FNA needle in sensitivity and adequacy for the diagnosis of SPLs. As described above, estimated pooled ORs in our meta‐analysis suggested the lower sensitivity in small SPLs was less prominent when a 25‐gauge needle was used (Figure 5a), as compared to studies without the use of a 25‐gauge needle. However, there has been no head‐to‐head comparative study of 25‐ and 22‐gauge needles in small SPLs. Furthermore, effectiveness of 25‐gauge FNB needles has been reported in SPLs,^45^, ^46^, ^47^ though one study did suggest sensitivity and adequacy decreased in small SPLs even using a 25‐gauge FNB needle.^47^ A prospective comparative study is warranted to elucidate whether a 25‐gaguge FNA or FNB needle would provide better diagnostic yield in small SPLs or not.

### The needle type and suction method

A recent RCT revealed a fork‐tip or Franseen FNB needle with no suction or slow pull suction provided the best accuracy and cellularity in SPLs.^42^ However, as described above, in our meta‐analysis, neither EUS‐FNB nor slow pull method appeared to increase sensitivity of EUS‐TA for small SPLs but recently various FNB needles with different designs and sizes are commercially available. Mie et al^48^ retrospectively compared three needles (22‐gauge FNA needle, 20‐gauge forward‐bevel FNB needle, and 22‐gauge Franseen needle) in small (<20 mm) SPLs and found the accuracy of the Franseen needle was 85.7%, compared to 92.7% with the FNA needle and 97.0% with the forward bevel FNB needle (*p* = 0.10). They speculated the Franseen geometry might make the needle puncture of small SPLs difficult, rather than the sharp tip of the other two needles. Itoi et al^49^ reported the size and type of FNA needles as well as the scope angulation affected the needle advancement resistance in an experimental study, and the resistance of the needle advancement can affect the diagnostic yield especially in cases with small lesions. Recent FNB needles have various features to increase cellularity of the specimen, and the resistance at needle advancement should be evaluated in these new FNB needles. We previously reported the slow pull method was associated with the better diagnostic yield in a 25‐gauge FNA needle^50^ and FNB needles.^51^ Although the use of slow pull method was not associated with the better diagnostic yield in our meta‐analysis, the best suction technique for small SPLs remains to be clarified.

### ROSE and the number of passes

The number of passes might affect the diagnostic yield of EUS‐TA, too. Per‐pass sensitivity analyses revealed the cumulative sensitivity was significantly higher in SPLs of >20 mm, when a 22‐gauge FNA needle was used.^31^ While the sensitivity reached a plateau at 93% after four passes in SPLs of >20 mm, the sensitivity after four passes was only 77% and increased up to 82% after six passes in SPLs of <20 mm, suggesting the increased number of passes might be necessary in small SPLs. Interestingly, a center‐based analysis revealed that sensitivity of EUS‐FNA did not differ by the size of SPLs in one center, but the sensitivity was significantly higher in SPLs of >20 mm in the other center, though its reason was unclear. In recent studies using 22‐gauge Franseen needles, two passes of EUS‐FNB appeared to be sufficient to obtain histological core and reach the diagnosis of SPLs as opposed to three to four passes of a 22‐gauge FNA needle,^39^, ^52^ but it is still unknown whether this is also true in the subgroup of small SPLs or not.

The role of ROSE during EUS‐TA is controversial, too. A recent meta‐analysis^53^ revealed ROSE did not improve the diagnostic yield of EUS‐FNA for SPLS. In our meta‐analysis focusing on the size of SPLs, the presence of ROSE did not seem to increase sensitivity in small SPLs, either, with an OR of 1.59 and 2.10 in studies with and without ROSE. Despite its high specificity, a relatively low negative predictive value is still a problem in EUS‐TA. In SPLs of <30 mm, repeat EUS‐FNA up to three sessions increased sensitivity from 68% to 92%.^54^ Thus, repeat EUS‐FNA after non‐diagnostic or inconclusive results is recommended, and ROSE might have a role after non‐diagnostic initial EUS‐FNA.^55^ The role of ROSE seems to decrease in the era of EUS‐FNB as shown in a recent RCT,^56^ but we still need further evidences about ROSE or macroscopic on‐site evaluation^57^ in a selected population such as small SPLs or negative initial EUS‐TA.

### Safety

In our literature review, comparative data on adverse events according to the lesion size are scarce. One single center retrospective study analyzed risk factors of adverse events of EUS‐TA.^58^ The adverse event rate was 3.4 % and SPLs of <20 mm was one of the predictive factors for adverse events with an OR of 18.48 (95% CI, 3.55–96.17). Another predictive factor was pancreatic neuroendocrine neoplasms (pNEN) with an OR of 36.50. A recent study of EUS‐TA using a 25‐gauge FNA needle also described that pancreatitis developed in 2 of 61 (3.3%) in SPLs of <15mm and 0/102 in SPLs of 15–25 mm, and both cases who developed pancreatitis had a diagnosis of pNEN.^59^ The risk of pancreatitis after EUS‐TA for pNEN was also reported in a multicenter retrospective study, too.^60^ Thus, EUS‐TA for small SPLs, especially pNEN, seems to have a high risk of adverse events and need caution. It is still unknown whether a specific needle or technique can reduce the risk of AE in patients with small SPLs.

### Unanswered questions and future research

Early diagnosis of pancreatic cancer is essential to improve its dismal prognosis, and the diagnosis of pancreatic cancer at sub‐centimeter size is necessary to achieve long‐term survival after curative resection.^61^ It is well known EUS can detect small SPLs than CT, and EUS‐TA is now established as the diagnostic procedure for SPLs.^5^ However, in our meta‐analysis, the diagnostic yield of EUS‐TA for small (<20 mm) SPLs is not satisfactory. Our subgroup analysis suggested the use of a 25‐gauge needle may mitigate the risk of non‐diagnostic EUS‐TA for small SPLs. However, in clinical practice, additional genome profiling is increasingly performed for pancreatic cancer. A recent study showed both a 25‐gauge FNA needle and a 19‐ or 22‐gauge FNB needle achieve sensitivity of 100% in diagnosing pancreatic cancer, but the adequate specimen for genome profiling was obtained only in 14% by a 25‐gauge FNA needle as compared to 78% by FNB needles.^62^ Due to the increased utilization of neoadjuvant chemotherapy in small resectable pancreatic cancer, the initial EUS‐TA prior to neoadjuvant chemotherapy might be the only opportunity to obtain undamaged specimens fit for genome profiling in small SPLs. Thus, it should be explored how we can increase the yield of genome profiling in small SPLs since EUS‐TA for resectable pancreatic cancer also has a risk of adverse events, including needle tract seeding.^63^ In general, a smaller needle with fewer passes is preferred to reduce the risk of needle tract seeding, but the evidence is still lacking about the risk factor for needle tract seeding. A large cohort study of EUS‐TA in resectable pancreatic cancer is mandatory since the tumor seeding rate is relatively low.^60^

In summary, the diagnostic yield of EUS‐TA for small SPLs still needs improvement. Since most studies included in our analysis are retrospective and heterogeneous with a high risk of bias, further prospective studies focusing on the diagnostic yield, the genomic yield and adverse events by EUS‐TA for small SPLs are warranted to clarify the best needle and technique.

## CONFLICT OF INTEREST

Yousuke Nakai receivedresearch grant from Boston Scientific Japan, Fujifilm Corporation, HOYA Corporation, Medico's Hirata and honoraria from Boston Scientific Japan, Fujifilm Corporation, Medico's Hirata, Medtronic, Olympus Corporation. Hirofumi Kogure received honoraria from Boston Scientific Japan, Fujifilm Corporation, Medico's Hirata, and Olympus Corporation. Mitsuhiro Fujishir received research grant from Fujifilm Corporation, HOYA Corporation, Olympus Corporation and honoraria from Fujifilm Corporation and Olympus Corporation. Yousuke Nakai is an associate editor of digestive endoscopy.

## FUNDING INFORMATION

None.

## Supporting information


**Supplementary Figure 1**. Funnel plots to examine potential publication bias in odds ratio. The x‐axis represents odds ratio, and the y‐axis displays the standard error of log (odds ratio). a. Comparison of adequacy between lesions of <20 mm and >20 mm. b. Comparison of sensitivity between lesions of <30 mm and >30 mm. c. Comparison of sensitivity between lesions of <20 mm and >20 mm. d. Comparison of sensitivity between lesions of <10 mm and >10 mm. e. Comparison of accuracy between lesions of <30 mm and >30 mm. f. Comparison of accuracy between lesions of <20 mm and >20 mm. g. Comparison of accuracy between lesions of <10 mm and >10 mm.
