# Supplementary figures and images for: Endoscopic ultrasonography‐guided tissue acquisition for small solid pancreatic lesions: Does the size matter?
Source: DEN Open. 2021 Sep 28;2(1):e52. doi: 10.1002/deo2.52 (PMC8828213; doi:10.1002/deo2.52)

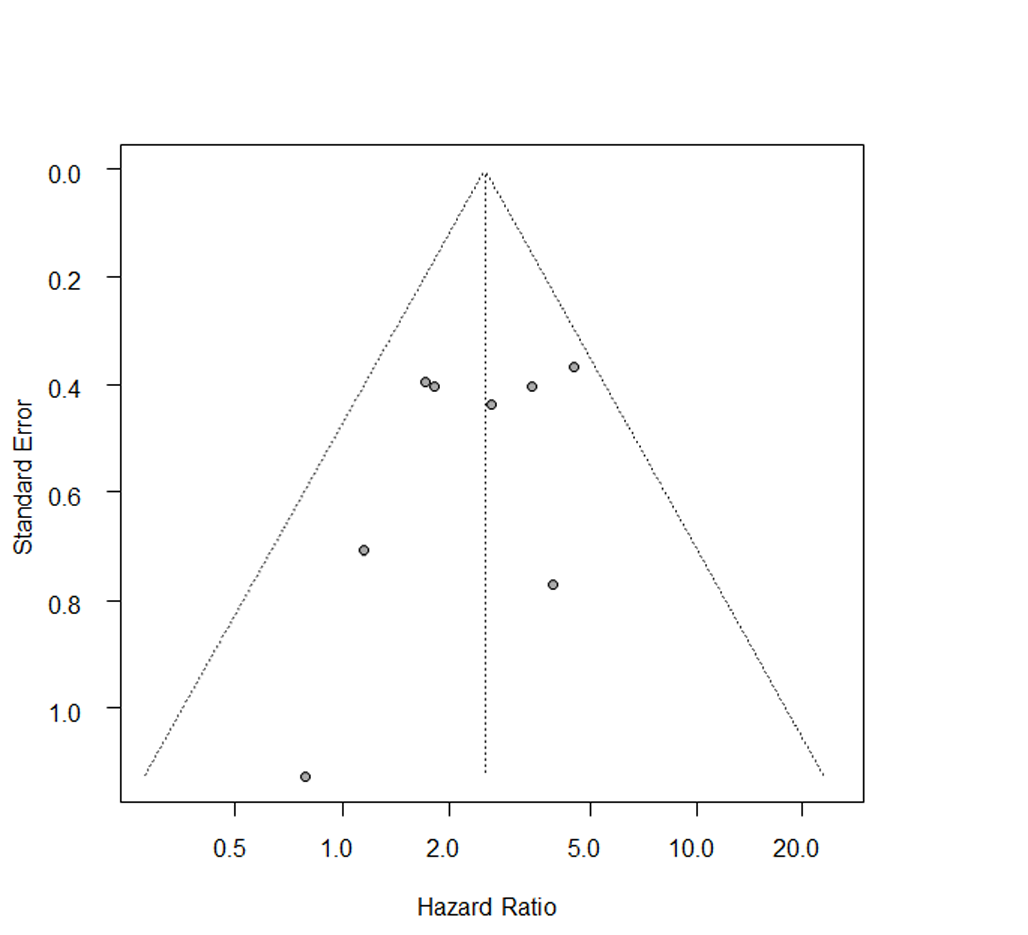

Supplement: Supplementary file 1 — Supplementary Figure 1. Funnel plots to examine potential publication bias in odds ratio. The x‐axis represents odds ratio, and the y‐axis displays the standard error of log (odds ratio). a. Comparison of adequacy between lesions of <20 mm and >20 mm. b. Comparison of sensitivity between lesions of <30 mm and >30 mm. c. Comparison of sensitivity between lesions of <20 mm and >20 mm. d. Comparison of sensitivity between lesions of <10 mm and >10 mm. e. Comparison of accuracy between lesions of <30 mm and >30 mm. f. Comparison of accuracy between lesions of <20 mm and >20 mm. g. Comparison of accuracy between lesions of <10 mm and >10 mm. [file DEO2-2-e52-s001.zip › deo252-sup-0001-FigureS1a.tif]

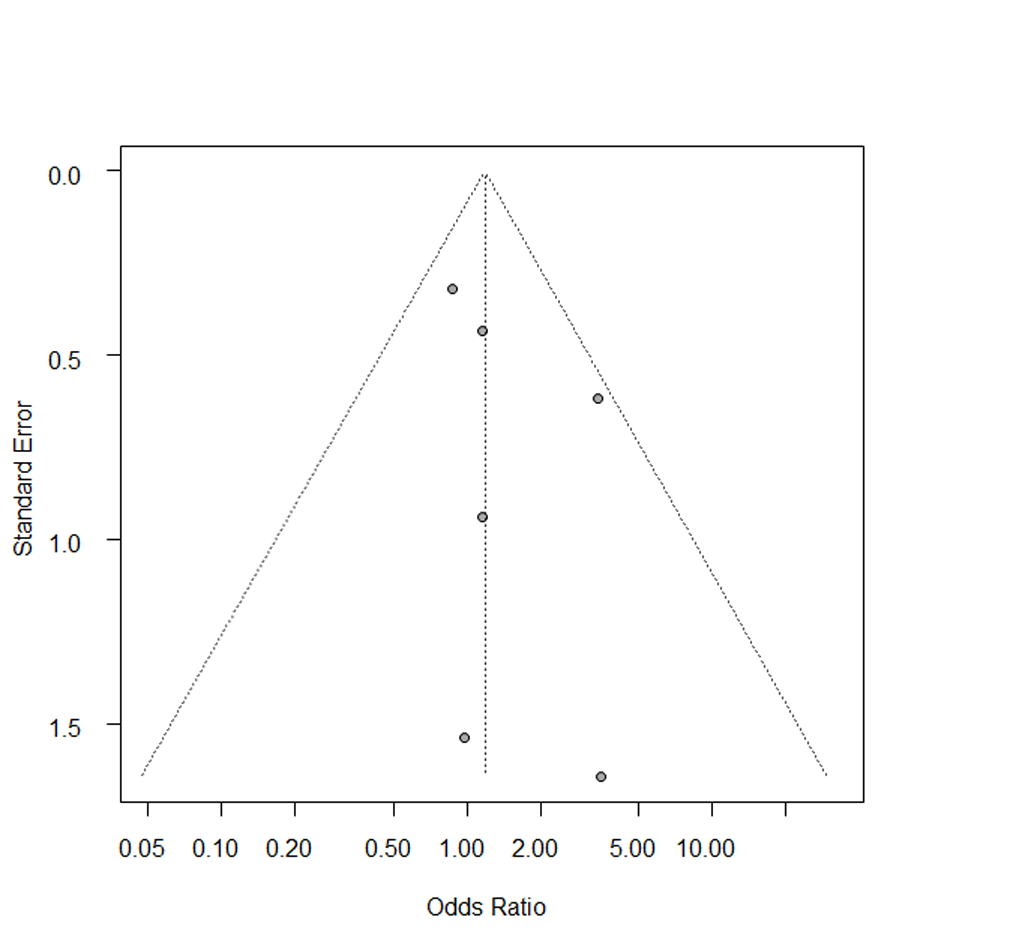

Supplement: Supplementary file 1 — Supplementary Figure 1. Funnel plots to examine potential publication bias in odds ratio. The x‐axis represents odds ratio, and the y‐axis displays the standard error of log (odds ratio). a. Comparison of adequacy between lesions of <20 mm and >20 mm. b. Comparison of sensitivity between lesions of <30 mm and >30 mm. c. Comparison of sensitivity between lesions of <20 mm and >20 mm. d. Comparison of sensitivity between lesions of <10 mm and >10 mm. e. Comparison of accuracy between lesions of <30 mm and >30 mm. f. Comparison of accuracy between lesions of <20 mm and >20 mm. g. Comparison of accuracy between lesions of <10 mm and >10 mm. [file DEO2-2-e52-s001.zip › deo252-sup-0001-FigureS1b.tif]

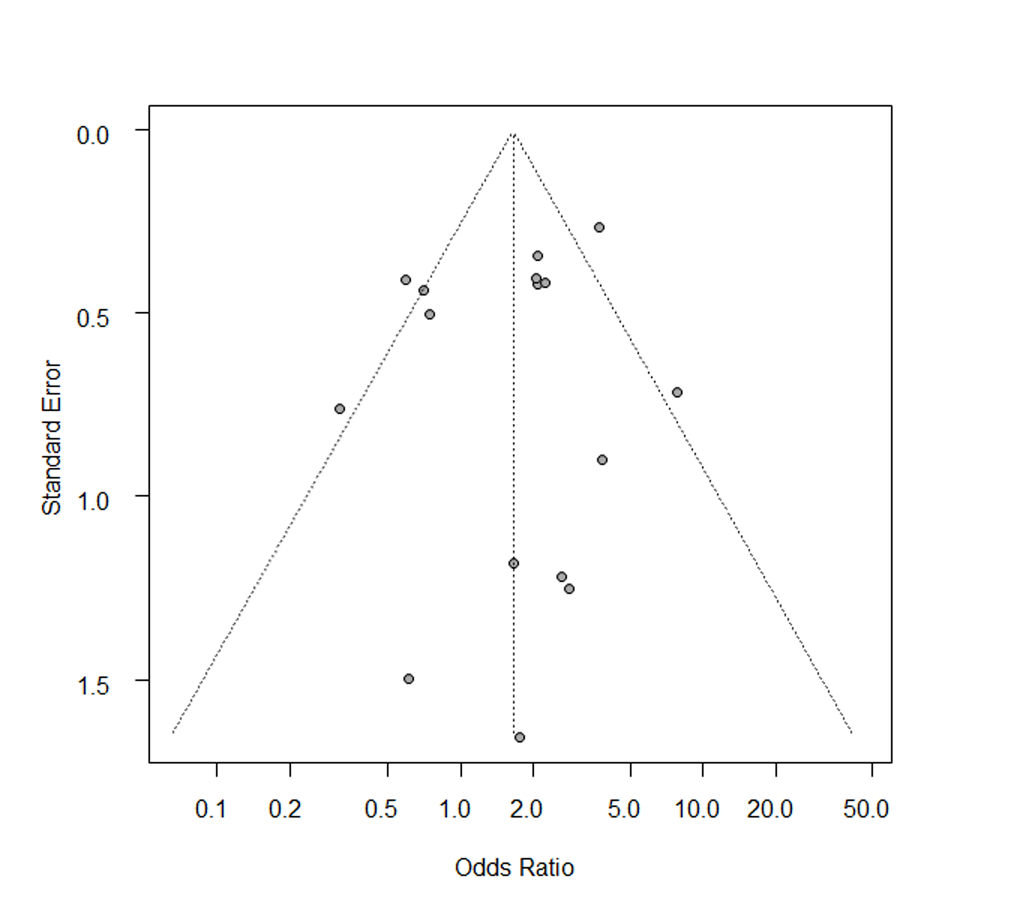

Supplement: Supplementary file 1 — Supplementary Figure 1. Funnel plots to examine potential publication bias in odds ratio. The x‐axis represents odds ratio, and the y‐axis displays the standard error of log (odds ratio). a. Comparison of adequacy between lesions of <20 mm and >20 mm. b. Comparison of sensitivity between lesions of <30 mm and >30 mm. c. Comparison of sensitivity between lesions of <20 mm and >20 mm. d. Comparison of sensitivity between lesions of <10 mm and >10 mm. e. Comparison of accuracy between lesions of <30 mm and >30 mm. f. Comparison of accuracy between lesions of <20 mm and >20 mm. g. Comparison of accuracy between lesions of <10 mm and >10 mm. [file DEO2-2-e52-s001.zip › deo252-sup-0001-FigureS1c.tif]

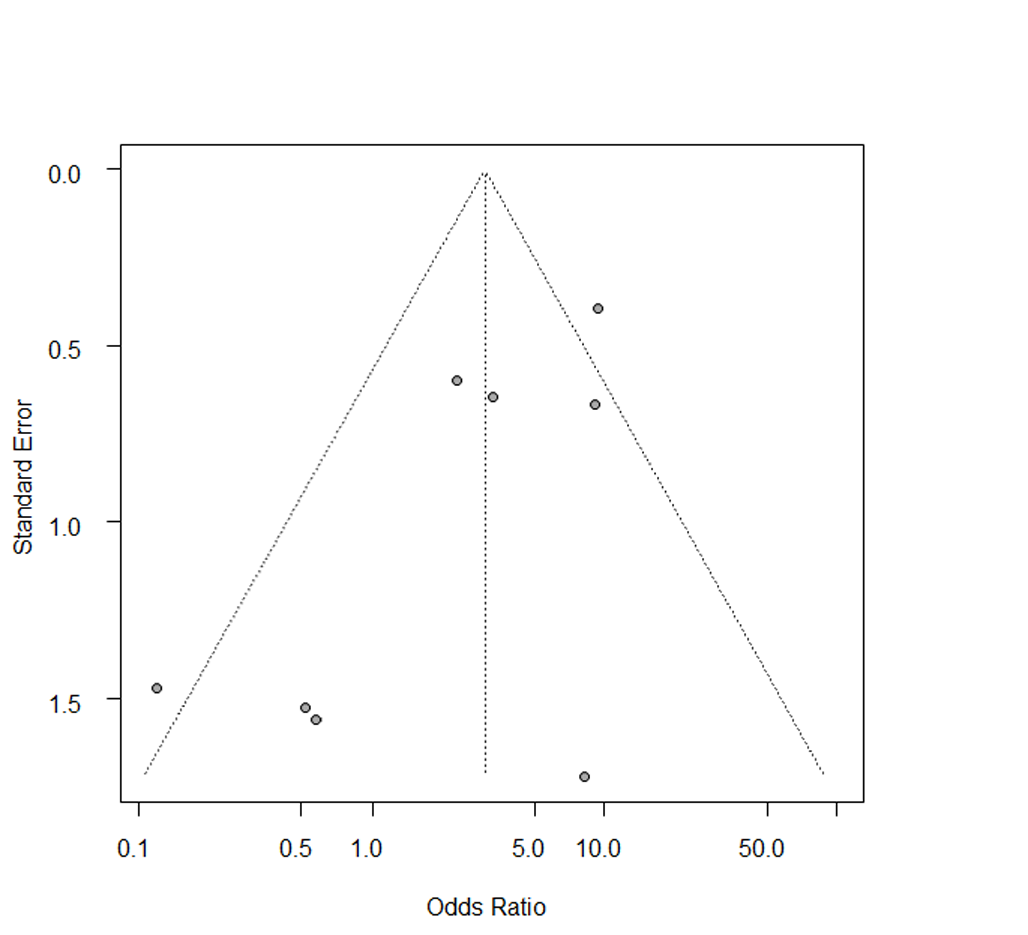

Supplement: Supplementary file 1 — Supplementary Figure 1. Funnel plots to examine potential publication bias in odds ratio. The x‐axis represents odds ratio, and the y‐axis displays the standard error of log (odds ratio). a. Comparison of adequacy between lesions of <20 mm and >20 mm. b. Comparison of sensitivity between lesions of <30 mm and >30 mm. c. Comparison of sensitivity between lesions of <20 mm and >20 mm. d. Comparison of sensitivity between lesions of <10 mm and >10 mm. e. Comparison of accuracy between lesions of <30 mm and >30 mm. f. Comparison of accuracy between lesions of <20 mm and >20 mm. g. Comparison of accuracy between lesions of <10 mm and >10 mm. [file DEO2-2-e52-s001.zip › deo252-sup-0001-FigureS1d.tif]

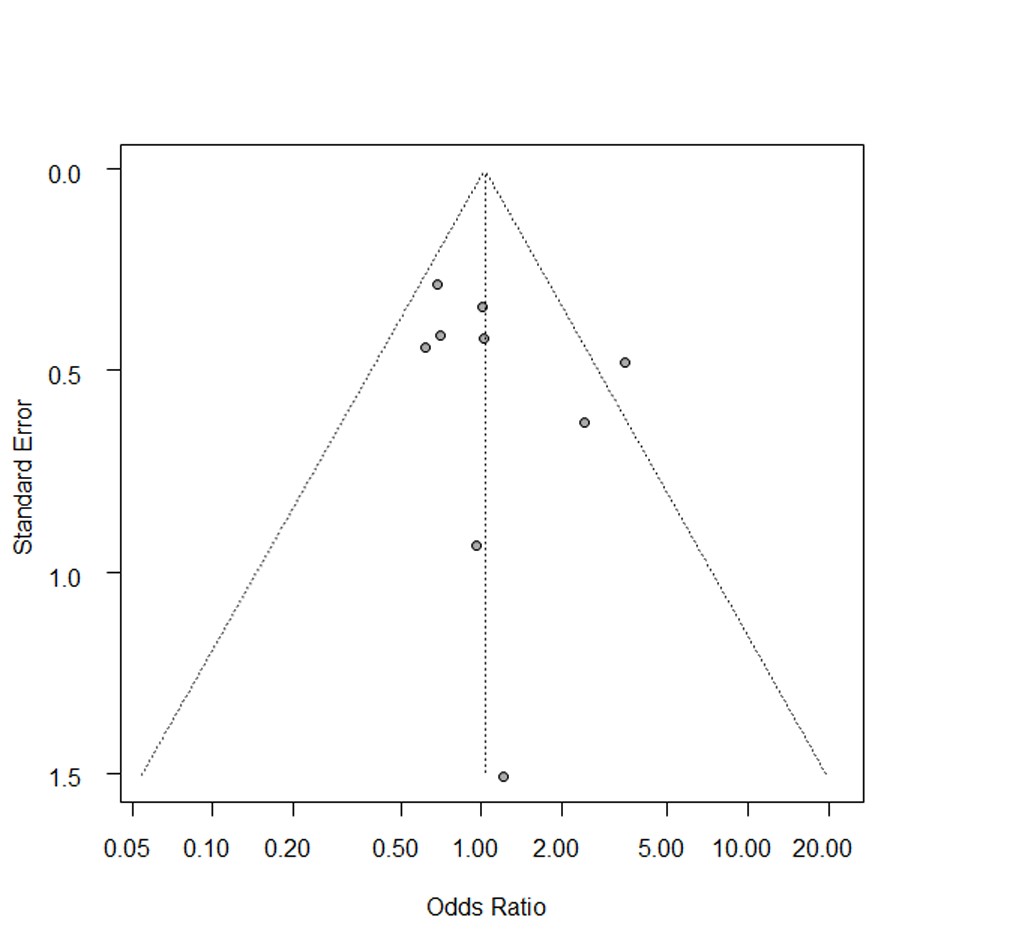

Supplement: Supplementary file 1 — Supplementary Figure 1. Funnel plots to examine potential publication bias in odds ratio. The x‐axis represents odds ratio, and the y‐axis displays the standard error of log (odds ratio). a. Comparison of adequacy between lesions of <20 mm and >20 mm. b. Comparison of sensitivity between lesions of <30 mm and >30 mm. c. Comparison of sensitivity between lesions of <20 mm and >20 mm. d. Comparison of sensitivity between lesions of <10 mm and >10 mm. e. Comparison of accuracy between lesions of <30 mm and >30 mm. f. Comparison of accuracy between lesions of <20 mm and >20 mm. g. Comparison of accuracy between lesions of <10 mm and >10 mm. [file DEO2-2-e52-s001.zip › deo252-sup-0001-FigureS1e.tif]

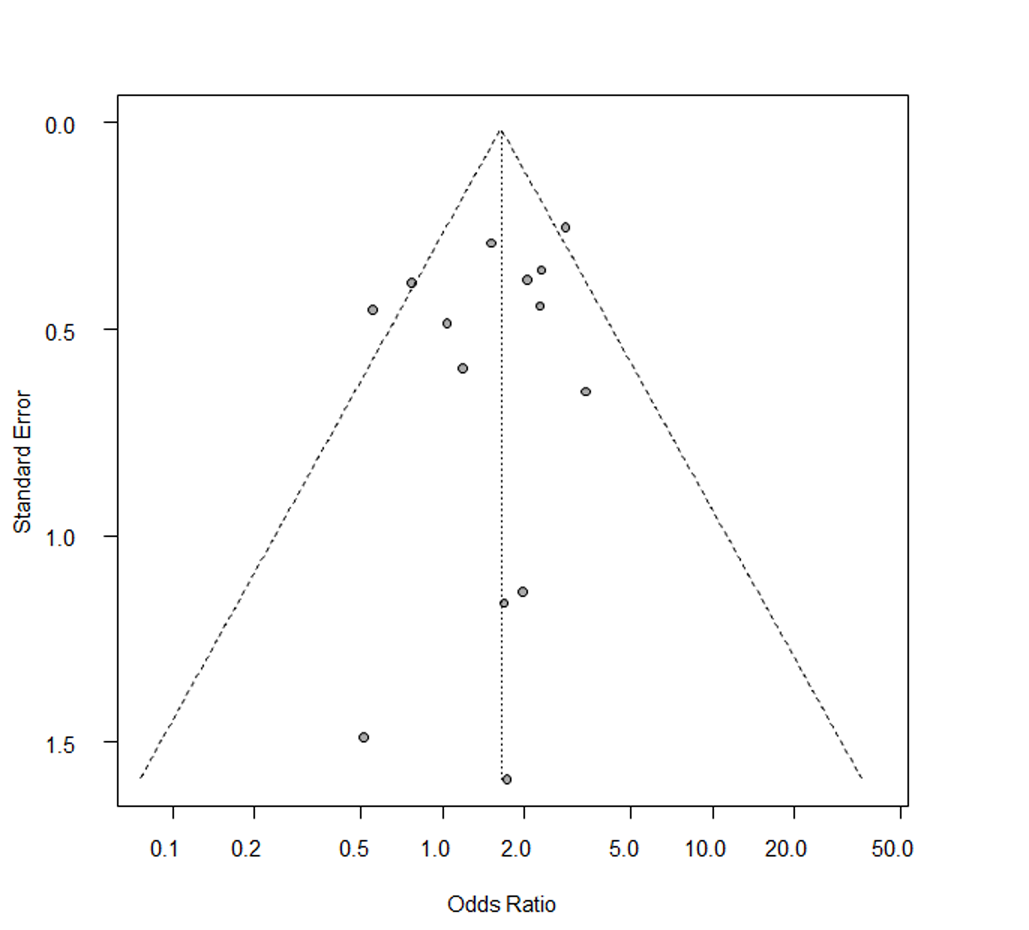

Supplement: Supplementary file 1 — Supplementary Figure 1. Funnel plots to examine potential publication bias in odds ratio. The x‐axis represents odds ratio, and the y‐axis displays the standard error of log (odds ratio). a. Comparison of adequacy between lesions of <20 mm and >20 mm. b. Comparison of sensitivity between lesions of <30 mm and >30 mm. c. Comparison of sensitivity between lesions of <20 mm and >20 mm. d. Comparison of sensitivity between lesions of <10 mm and >10 mm. e. Comparison of accuracy between lesions of <30 mm and >30 mm. f. Comparison of accuracy between lesions of <20 mm and >20 mm. g. Comparison of accuracy between lesions of <10 mm and >10 mm. [file DEO2-2-e52-s001.zip › deo252-sup-0001-FigureS1f.tif]

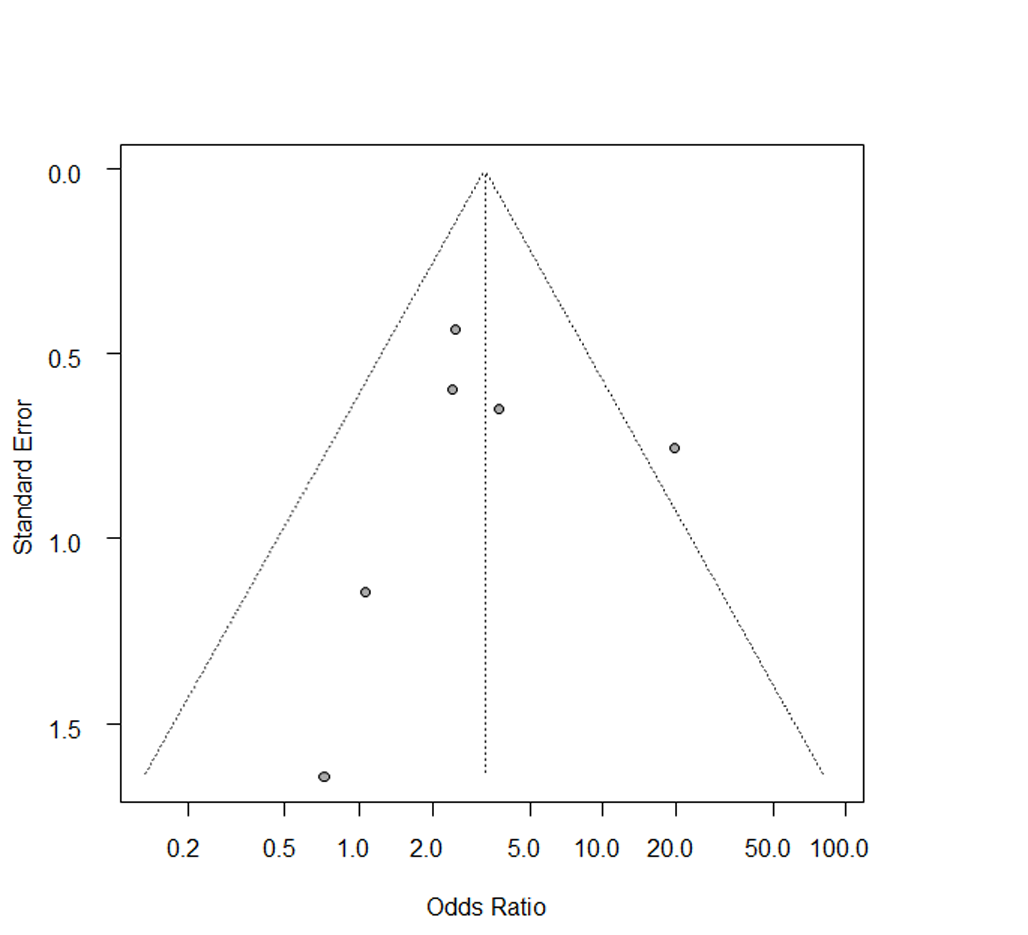

Supplement: Supplementary file 1 — Supplementary Figure 1. Funnel plots to examine potential publication bias in odds ratio. The x‐axis represents odds ratio, and the y‐axis displays the standard error of log (odds ratio). a. Comparison of adequacy between lesions of <20 mm and >20 mm. b. Comparison of sensitivity between lesions of <30 mm and >30 mm. c. Comparison of sensitivity between lesions of <20 mm and >20 mm. d. Comparison of sensitivity between lesions of <10 mm and >10 mm. e. Comparison of accuracy between lesions of <30 mm and >30 mm. f. Comparison of accuracy between lesions of <20 mm and >20 mm. g. Comparison of accuracy between lesions of <10 mm and >10 mm. [file DEO2-2-e52-s001.zip › deo252-sup-0001-FigureS1g.tif]
